# Journal of Neurodevelopmental Disorders reviewer acknowledgement 2013

**DOI:** 10.1186/1866-1955-6-2

**Published:** 2014-01-30

**Authors:** Joseph Piven

**Affiliations:** 1 Carolina Institute for Developmental Disabilities, University of North Carolina at Chapel Hill, 100 Renee Lynne Court, Carrboro, NC 27510, USA

## Abstract

**Contributing reviewers:**

We thank all of our reviewers who have contributed to the journal in volume 5 (2013). High quality and timely reviews are critical to the overall quality of the journal. We are committed to providing a unique and important outlet for scholarship regarding neurodevelopmental disorders and are indebted to the outstanding reviewers who have contributed their time over the last year in helping us to reach this goal.

## 

Elizabeth Aylward

United States of America

Ben A. Barres

United States of America

Carrie Bearden

United States of America

Karl Bechter

Germany

Dorothy Bishop

United Kingdom

Nash Boutros

United States of America

William Brown

United States of America

Melissa Carter

Canada

Guillermo Cecchi

United States of America

Tony Charman

United Kingdom

Naseem Choudhury

United States of America

Andrea Contestabile

Italy

Jacqueline Crawley

United States of America

Leopold Curfs

Netherlands

Arnab Datta

Singapore

Paola Dazzan

United Kingdom

Gabriel Dichter

United States of America

Manny DiCicco-Bloom

United States of America

Anastasia Dimitropoulos

United States of America

Magdalena Dziembowska

Poland

Christine Ecker

United Kingdom

Jed Elison

United States of America

Susan Ellis Weismer

United States of America

Michela Fagiolini

United States of America

Jin Fan

United States of America

Emily Farran

United Kingdom

Faraz Farzin

United States of America

Laura Flores-Sarnat

Canada

Susan Folstein

United States of America

Tim Fosker

United Kingdom

Louise Gallagher

Ireland

Matthew Goodwin

United States of America

Brian Haas

United States of America

Paul Hagerman

United States of America

Randi Hagerman

United States of America

Brian Hallahan

Ireland

Tarik Haydar

United States of America

David Hessl

United States of America

Tony Holland

United Kingdom

Jamie Horder

United Kingdom

Patricia Howlin

United Kingdom

Kimberly Huber

United States of America

Stephan Huijbregts

Netherlands

Shafali Jeste

United States of America

Peng Jin

United States of America

Klaus Kessler

United Kingdom

Alexandra Key

United States of America

Bonita Klein-Tasman

United States of America

Ami Klin

United States of America

Kami Koldewyn

United Kingdom

Shanna Kralovic

United States of America

Meng-Chuan Lai

United Kingdom

Jasmin Lalonde

United States of America

Hayley Leonard

United Kingdom

Amir Levine

United States of America

Ovsanna Leyfer

United States of America

Molly Losh

United States of America

Eva Loth

United Kingdom

Kristen Lyall

United States of America

Elena Maestrini

Italy

Marsha Mailick

United States of America

Christian Marshall

Canada

Marilee Martens

United States of America

James McPartland

United States of America

Suzie Miller

Australia

Anna Victoria Molofsky

United States of America

Jeff Munson

United States of America

Michael Murias

United States of America

David Nelson

United States of America

Giovanni Neri

Italy

Linda Noble

United States of America

Courtenay Norbury

United Kingdom

Richard Nowakowski

United States of America

Lindsay Oberman

United States of America

Joachim Oertel

Germany

Jose Luis Olmos Serrano

United States of America

Lucy Osborne

Canada

Jorge Palop

United States of America

Katherine Perdue

United States of America

David Picketts

Canada

Andrew Pieper

United States of America

Karen Pierce

United States of America

Daniela Plesa Skwerer

United States of America

Melanie Porter

Australia

Gudrun Rappold

Germany

Brian Rash

United States of America

Wilburn Reddick

United States of America

Sean Redmond

United States of America

Allan Reiss

United States of America

Mabel Rice

United States of America

Timothy Roberts

United States of America

Jacqui Rodgers

United Kingdom

Miguel Ángel Romero-Munguía

Mexico

Noah Sasson

United States of America

Kimberly Scearce-Levie

United States of America

Gaia Scerif

United Kingdom

Sarah Schoen

United States of America

Robert Schultz

United States of America

Frederick Shic

United States of America

Barbara Shinn-Cunningham

United States of America

Jeremy Silverman

United States of America

Nancy Snidman

United States of America

Marjorie Solomon

United States of America

Daniel Stahl

United Kingdom

Catherine Stamoulis

United States of America

Julia Stephen

United States of America

Hanna Stevens

United States of America

Yujiao Sun

United States of America

Elyse Sussman

United States of America

James Sutcliffe

United States of America

Peter Szatmari

Canada

Marc J. Tasse

United States of America

Mark Taylor

United Kingdom

Erik Ullian

United States of America

Therese van Amelsvoort

Netherlands

Jean-Michel Verdier

France

Willem M.A. Verhoeven

Netherlands

Annick Vogels

Belgium

Jennifer Wagner

United States of America

Le Wang

United States of America

Sara Webb

United States of America

Rosanna Weksberg

Canada

Corrine Welt

United States of America

Jeffrey Wood

United States of America

Zhengang Yang

China

Ryan Yuen

Canada

Lonnie Zwaigenbaum

Canada

